# The Impact of Hypnotic Suggestions on Reaction Times in Continuous Performance Test in Adults with ADHD and Healthy Controls

**DOI:** 10.1371/journal.pone.0126497

**Published:** 2015-05-11

**Authors:** Maarit Virta, Seppo Hiltunen, Markus Mattsson, Sakari Kallio

**Affiliations:** 1 Division of Cognitive and Neuropsychology, Institute of Behavioural Sciences, University of Helsinki, Helsinki, Finland; 2 The Helsinki University Centre for Research and Development of Higher Education, Institute of Behavioural Sciences, University of Helsinki, Helsinki, Finland; 3 Department of Cognitive Neuroscience and Philosophy, School of Bioscience, University of Skövde, Skövde, Sweden; 4 Centre for Cognitive Neuroscience, University of Turku, Turku, Finland; National Center of Neurology and Psychiatry, JAPAN

## Abstract

Attention is one of the key factors in both hypnotic processes and patients with ADHD. In addition, the brain areas associated with hypnosis and ADHD overlap in many respects. However, the use of hypnosis in ADHD patients has still received only minor attention in research. The main purpose of the present work was to investigate whether hypnosis and hypnotic suggestions influence the performance of adult ADHD (n = 27) and control participants (n = 31) in the continuous performance test (CPT). The hypnotic susceptibility of the participants was measured by the Harvard Group Scale of Hypnotic Susceptibility (HGSHS:A) and the attentional task was a three minute long auditory version of the CPT. The CPT task was administered four times: before hypnosis (CPT1), after a hypnotic induction (CPT2), after suggestions about speed and accuracy (CPT3), and after the termination of hypnosis (CPT4). The susceptibility of the groups measured by HGSHS:A did not differ. There was a statistically significant decrease in reaction times in both ADHD and control groups between CPT2 and CPT3. The differences between CPT1 and CPT2, even though non-significant, were different in the two groups: in the ADHD group reaction times decreased whereas in the control group they increased. Both groups made very few errors in the short CPT. This study indicates that hypnotic suggestions have an effect on reaction times in the sustained attention task both in adult ADHD patients and control subjects. The theoretical and clinical implications are discussed.

## Introduction

Attention deficit hyperactivity disorder (ADHD) is a developmental neurobiological disability characterized by deficits in attention and executive functions and/or symptoms of hyperactivity and impulsivity [1]. It emerges in childhood and often continues into adulthood. The prevalence of ADHD in adults has been estimated to be 4.4% [2]. Current research suggests that there are alterations in structure and dysfunction in activity in multiple neuronal systems and networks [3–5]. In particular, a decrease in total cerebral and cerebellar volume is found. The dysfunctional areas include anterior cingulate cortex, frontostriatal circuitry, cerebellum, temporoparietal lobes, basal ganglia, thalamus, amygdala, hippocampus and corpus callosum (for review see e.g. [4–6]). There is evidence about alterations in activity in attention networks [3,7,8], default mode network [3,7,8], salience network [7], sensorimotor systems [3], affective network [8] and executive control network [7], and dysconnectivity of default mode network [9].

Adult ADHD typically manifests as deficits in organizing, prioritizing, and activating oneself to work; focusing, sustaining, and shifting attention to tasks; regulating alertness, sustaining effort, and processing speed; managing frustration and modulating emotions; utilizing working memory and accessing recall; and/or monitoring and self-regulating action [10]. Some, but not all, adults with ADHD also have hyperactivity, which may be limited to feelings of restlessness. These deficits generally cause marked impairment in the educational, occupational, and social functioning of the adults with ADHD.

Hypnosis can be defined in the following way: “Operationally, hypnosis refers to a change in baseline mental activity after an induction procedure and typically experienced at a subjective level as an increase in absorption, focused attention, disattention to extraneous stimuli and a reduction in spontaneous thought” [11]. Although the brain mechanisms associated with hypnosis are still unclear, the importance of the prefrontal cortex and anterior cingulate cortex has been proposed in several studies [12–15]. Hoeft and colleagues [13] argue that altered functional connectivity (see also [16]) between executive-control regions and the salience network may underlie hypnotizability. There are some studies that show decreased activity in the default mode network during hypnosis [14,17,18] and increased activity in the prefrontal attentional system [17].

Attention is a key factor in hypnotic processes [19,20]. The Stroop task is one of the most studied tasks in the attention research and it is also used to study behavioral effects of hypnotic suggestions. There is strong evidence that the Stroop effect is reduced or even eliminated by using posthypnotic suggestions [21–24]. In these studies the effect was achieved only in highly suggestible individuals (HSIs) but in one study [25] the reduction of the Stroop effect was also shown in low suggestible individuals (LSIs) although to a lesser extent. Furthermore, non-hypnotic suggestions have also reduced the Stroop effect [22]. The Stroop effect is thought to reflect highly automatic cognitive processes [26,27]. There is also preliminary evidence that with suggestions more controlled processes can be shifted to more automatic [28].

When a relevant event occurs at a relatively slow rate over a prolonged period there is a need for sustaining attention. The most commonly used tests in both clinical practice and research to study sustained attention are continuous performance tests (CPTs). CPTs are considered to be reliable and sensitive measures of attention and attentional system dysfunction [29,30]. There are several versions of CPTs but usually the participants are required to detect a target stimulus among nontargets. The task usually takes 10–30 minutes [31] and the stimuli are usually presented visually. Adults with ADHD make more omission and commission errors than controls [29,32–36]. In reaction times there are usually no or negligible differences [29,35,36]. However, the results are equivocal and controversial with there being evidence also for both slower reaction times [32,34] and marginally faster reaction times [33] in adults with ADHD.

In the Kallio et al study [37] several attentional or executive tasks were used. They found that in neutral hypnosis (i.e. without specific suggestions) reaction times increased in a simple reaction time task and vigilance task in both LSIs and HSIs compared to no hypnosis condition.

The aim of this study was to test the influence of hypnosis, hypnotizability, and hypnotic suggestions on the performance of ADHD and control participants in the CPT. Attention is a common key factor in both hypnosis and ADHD and, furthermore, there is clear overlap of the significant brain areas (e.g. frontal areas, anterior cingulate cortex) and alterations in functional networks (especially default mode network). On the basis of previous studies we hypothesize that in controls the reaction times will be slower after induction (neutral hypnosis) and faster after hypnotic suggestions. No predictions are made about between group differences (controls vs ADHD) due to the lack of previous studies.

## Methods

### Participants

Participants were recruited through advertisement in an ADHD magazine, in an adult ADHD internet discussion forum and by informing local physicians and clinics specialized in treating ADHD in adults. The control participants were recruited as above and from an e-mail list of psychology students. The inclusion criteria were as follows: (1) 18–45 years of age, (2) no diagnosis of psychosis or bipolar disorder, and (3) no current severe depression. Those for the ADHD group should also have ADHD diagnosis made by a physician and those for control group should not have an ADHD diagnosis or ADHD symptoms.

In total, 53 potential participants for the ADHD group and 36 for the control group contacted the researchers. Of the ADHD candidates, nine individuals were excluded for not meeting all the inclusion criteria, and 16 cancelled or discontinued participation. One participant did not respond behaviorally to given hypnotic suggestions in HGSHS:A and was excluded from the analysis. Of the control group, one candidate was excluded because she/he reported ADHD symptoms and three cancelled participation. We also excluded one participant from the analysis due to missing information in the CPT (technical problem or participant pressed button too lightly). Thus, there were a total of 27 participants in the ADHD group and 31 in the control group.

Demographic data of the two groups are presented in Table 1. The two groups did not differ (as analyzed by Chi-Square test) in gender [*χ* ^2^ (1) = 1.47, *p* = 0.25]. They differed, as analyzed by t-test or Chi-Square test/Fisher exact test in age [*t*(56) = -4.32, *p* < 0.001], in education [*χ^2* (1) = 6.82, *p* = 0.018, in work-status [*χ* ^2^ (1) = 4.93, *p* = 0.041], in ASRS score [*t*(56) = -15.89, *p* < 0.001], in SCL-90 sum score [*t*(56) = -5.69, *p* < 0.001] and in number of participants having psychiatric comorbidity [*χ* ^2^ (1) = 11.38, *p* < 0.001].

**Table 1:** Characteristics of the participants.

	ADHD	Controls
Participants (n)	27	31
Age: mean (range)	31.7 (22–45)	25.2 (19–45)
Gender: male/female (n)	9/18	6/25
Education: compulsory/additional (n) ^a^	5/22	0/31
Working or studying: yes / no (n) ^b^	23/4	31/0
Antidepressant medication (n)	3	1
Psychiatric comorbidity (n)	12	2
depression (n)	10	2
anxiety (n)	4	0
personality disorder (n)	2	0
ASRS score: mean (SD)	49.4 (9.3)	16.0 (6.7)
SCL-90 score: mean (SD)	161.4 (41.3)	114.7 (18.4)
HGSHS:A score: mean (SD) ^c^	7.15 (2.52)	6.42 (2.36)

Note

^a^ Compulsory = the participant had completed only lower secondary education (i.e. Finnish compulsory education)

^b^ Working/studying yes = the participant was working (at least a half-time job) or studying

^c^ Harvard Group Scale of Hypnotic Susceptibility, form A

The ADHD participants were requested—if possible—to be without ADHD medication during test sessions. 10 did not have any ADHD medication and 17 were medicated. Six of the medicated were unmedicated in both sessions. From the 11 medicated participants, seven of them took during first session methylphenidate, two took dextroamphetamine, one bupropioni and one methylphenidate and bupropioni. In the second session there were changes in two of them: one had ceased taking methylphenidate medication and one had changed methylphenidate to dextroamphetamine and bupropioni.

The study was approved by the Ethics Committee of the Helsinki University Central Hospital, Finland, and performed in accordance with the ethical standards laid down in the 1964 Declaration of Helsinki. All participants gave their written informed consent prior to participating in the study.

### Procedure

The participants attended two sessions: measurement of hypnotic susceptibility and the actual experimental session. They also completed questionnaires to ensure their suitability for the study and to explore the number of their symptoms. The questionnaires were either completed during the first session or submitted via mail beforehand. The questionnaires used were:

- *Questionnaire of background information*. In this questionnaire participants reported detailed information about education, work, health and medication.
- *World Health Organization's Adult ADHD Self-report Scale (ASRS)* [38]. ASRS is an 18-item scale reflecting the DSM-IV criteria for ADHD modified for adults.
- *Symptom Check List (SCL-90)* [39]. SCL-90 is a 90-item self-report scale for the measurement of psychiatric symptoms. Several subscales can be calculated. However, in this study the total score was used.

In the first session, hypnotic susceptibility was measured by using the Finnish version [40,41] of the Harvard Group Scale of Hypnotic Susceptibility, Form A (HGSHS:A) [42]. Some of the control subjects had already participated in a previous (currently unpublished) study for hypnotic susceptibility about one year earlier. These individuals participated only in the second session and filled out the above-mentioned questionnaires before the experimental task.

During the second session the participants were tested individually using a modification of CPT. The task was presented once in the four different experimental conditions. In each condition the task was to detect a target letter (30% of the stimuli) by pressing a button. Each CPT block contained 100 auditory stimuli (letters), the interstimulus-interval was 1800 ms and the whole block took three minutes. The acoustic intensity of the stimuli was adjusted individually to a comfortable level during the practice session because clinically many ADHD patients are hypersensitive to various stimuli. The target letter was different in each four conditions (A, I, U, or Y) and the order was counterbalanced between participants. First, participants participated in a short practice session to become familiar with the task. In the experimental session, the four conditions were: 1) before hypnosis (CPT1), 2) after a hypnotic induction, i.e. neutral hypnosis (CPT2), 3) after suggestions (CPT3), and 4) after the termination of hypnosis and suggestions (CPT4). The participants received identical instructions in all four CPT conditions: “After a while you will hear letters. Press the button as quickly and accurately as possible when you hear the target letter. The target letter is A/I/U/Y. The task begins now.” The reaction times for correct and incorrect reactions, and the number of correct hits and omission and commission errors were measured. The hypnotic induction was carried out in a structured way, whilst allowing for some personal modification (time to close the eyes). This induction consisted of eye fixation, relaxation and deepening of hypnosis by counting numbers, and took around 8 minutes to carry out. The hypnotic suggestions were administered after CPT2 was finished. The suggestions used in CPT3 condition (translated from Finnish) were:

*After a while*, *when the task begins*, *you are very attentive and quick*. *You hear the letters easily*. *You are focused and specifically hear the target letter*. *The target letter is A/I/U/Y*. *When the task begins you hear the target letter accurately and react quickly*. *Other sounds or things do not disrupt your concentration and you do not at all care about the other letters*. *You are very attentive and focused*, *and you react to the target letter by pressing the button very quickly*. *The impulse from your mind to your finger is instantaneous and you react very quickly and accurately*. *All your attention is focused on the target letter and you react very quickly and accurately*.

The whole procedure (preparation, induction, four CPT sessions, termination) lasted around 30 minutes. After CPT3 hypnotic reversal procedure was administered. It included counting numbers from 10 to 1 with suggestions about waking and returning to normal waking state with special emphasis on the normalization of attention and speed. A detailed description of the hypnotic procedure as a whole is available from the first author.

### Statistical analysis

A two-way mixed design repeated measures analysis of variance (2x4 rmANOVA) was carried out to investigate reaction time differences between the two groups. A fundamental assumption of rmANOVA is that of sphericity, defined as the equality of the variances of the differences between paired levels of the repeated measures factor. The assumption of sphericity was tested using Mauchly’s test, which is, however, known to lack power in small samples. For this reason, and to avoid type I errors, we applied the Greenhouse-Geisser correction to all the degrees of freedom of the F-tests. All degrees of freedom values in the results of rmANOVA are rounded to the nearest integer where applicable.

Using rmANOVA also requires the normality of residuals in all cells of the design. Normality assumptions were assessed using the Shapiro-Wilk test together with inspecting histograms and quantile-quantile-plots of the residuals. To ensure the validity of the results, all analyses were re-run after applying a 1/x transformation to all the reaction times. In addition, we re-ran the analyses using the median reaction time of each participant in each condition to ensure that the few long reaction times did not affect the results. None of our main conclusions were altered in these analyses.

The effect sizes of rmANOVA were quantified by partial eta squared. To better understand the differences in reaction times between the experimental conditions, four within-group planned comparisons were carried out using paired samples t-tests separately for the two experimental groups. Below, we report the p-values of these tests applying a Bonferroni correction for multiple comparisons and report the effect sizes (Cohen’s d) of the tests.

Omission errors on target letters were considered in the reaction time analysis as missing reaction time values. Omission errors and commission errors (faulty reactions) on other letters were analyzed together as the number of errors was low. Separate Poisson regression analyses were used to investigate error counts across groups in the four experimental conditions. In all Poisson regression models there were signs of overdispersion (the variance exceeding the mean). A scaling correction factor based on the Pearson chi-square goodness-of-fit statistic was therefore applied to the results [43]. In addition to the regression coefficients, the results of the likelihood ratio chi-square test are reported. This test indicates whether the model with the predictor variable fits the data better than the intercept-only model.

When investigating error counts within groups, Poisson regression could not be used due to the non-independence of the observations. Rather, a Generalized Estimating Equations (GEE) analysis was performed separately for both experimental groups, and additionally for the whole group of respondents. The experimental condition was used as the sole predictor, with number of errors as the dependent variable. A loglinear Poisson model with an unstructured correlation matrix of the repeated measures was fit to the data. A scaling correction factor for overdispersion was applied similarly to the Poisson regression analysis described above.

A significance level of 0.05 was used for all analysis. Applying the Bonferroni correction may produce p-values that exceed the value of 1. In such cases, we report the p-values as '1'. Further details on the statistical analyses can be obtained from the second or the third author via e-mail.

## Results

### Reaction times

**Fig 1:**
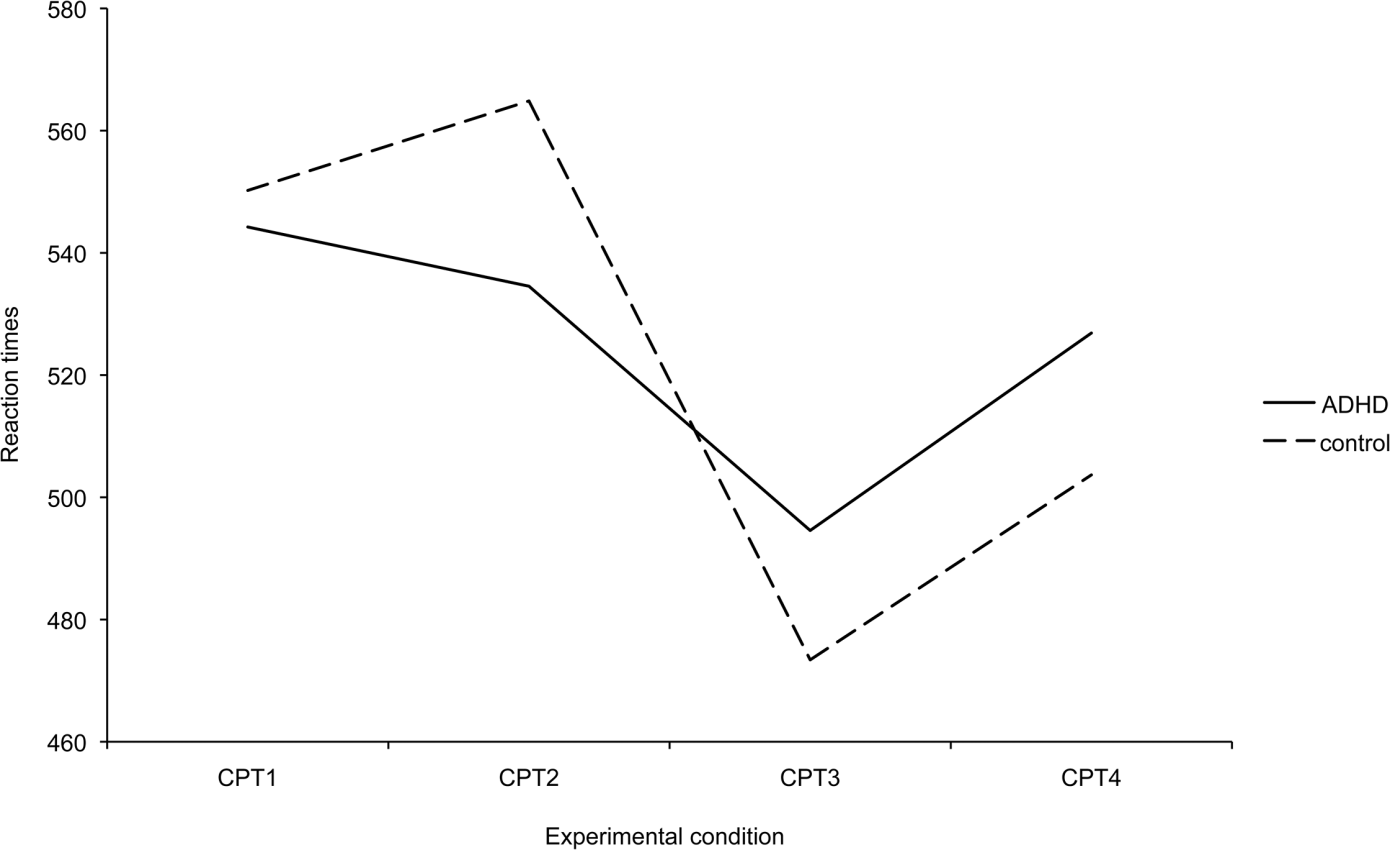
The mean reaction times in experimental conditions for ADHD patients and healthy control participants.

When comparing mean reaction times of the ADHD and control groups in the baseline experimental condition, i.e. CPT1, they did not differ significantly [*t*(56) = 0.23, *p* = 0.82]. To investigate whether the reaction times in the ADHD and control groups differed across test conditions, a 2x4 rmANOVA was carried out. The test showed that there was a significant test condition × group interaction in mean reaction times [*F*(3, 158) = 2.86, *p* = 0.042, partial eta squared = 0.05]. The result implies that the reaction time profiles across test conditions were different in the two groups (Table 2 and Fig 1).

**Table 2:** Reaction times and errors in ADHD and control groups at CPT1 (before hypnosis), CPT2 (after hypnotic induction), CPT3 (after speed and accuracy suggestions), and CPT4 (after hypnosis) test situations.

	ADHD (n = 27)				Controls (n = 31)			
	CPT1	CPT2	CPT3	CPT4	CPT1	CPT2	CPT3	CPT4
RT(sd)	544.2 (112.8)	534.5 (162.2)	494.6 (119.7)	526.9 (121.3)	550.2 (87.5)	564.9 (105.3)	473.4 (83.6)	503.7 (85.0)
ERR%	0.22 (0.51)	0.52 (1.19)	0.56 (1.05)	0.52 (1.19)	0.06 (0.25)	0.13 (0.43)	0.06 (0.25)	0.29 (0.59)
ERRORS	6	14	15	14	2	4	2	9
OMI/COM	2/4	9/5	8/7	8/6	0/2	2/2	1/1	0/9

Note.1

RT = mean reaction times and standard deviation

ERR% = percent of errors (omission and comission errors together)

ERRORS = total amount of errors of the group

OMI/COM = number of omission and commission errors of the group

These reaction time patterns were investigated in further detail using paired samples t-tests separately in the ADHD and the control group. CPT1 was compared with CPT2, CPT3 and CPT4, and CPT2 with CPT3. In the ADHD group, the mean reaction times did not differ between test conditions CPT1 and CPT2 [*t*(26) = 0.49, *p* = 1.00, *d* = 0.09], which they did between test conditions CPT2 and CPT3 [*t*(26) = 2.84, *p* = 0.034, *d* = 0.55]. The comparison between CPT1 and CPT3 resulted in a Bonferroni corrected p-value that approached statistical significance [*t*(26) = 2.53, *p* = 0.071, *d* = 0.49]. However, the effect size was moderate, with the mean reaction times differing by half a standard deviation. It is of interest to note that the difference CPT2-CPT3 was statistically significant, whereas the difference CPT1-CPT3 was only nearly so. This was due to subjects reacting differently to being exposed to conditions CPT2 and CPT3. When comparing CPT3 with the baseline measurement CPT1, the reaction times of eight participants increased and those of the others decreased. When comparing CPT3 with CPT2, there was less variation in the pattern of differences, with less increase in reaction times from CPT2 to CPT3. This resulted in greater variance in the difference scores in the comparison CPT1-CPT3, reflected in the greater p-value and the slightly smaller effect size than in the comparison CPT2-CPT3.

In the control group, the mean reaction times did not differ between test conditions CPT1 and CPT2 [*t*(30) = -1.11, *p* = 1.00, *d* = 0.20], whereas a statistically significant difference was noted between test conditions CPT1 and CPT3 [*t*(30) = 6.06, *p* < 0.001, *d* = 1.09] and CPT2 and CPT3 [*t*(30) = 5.61, *p* < 0.001, *d* = 1.01]. In both groups, the statistically significant difference between conditions CPT2 and CPT3 indicates that the hypnotic suggestions resulted in faster reaction times, over and above the effect of hypnotic induction.

The differences between CPT1 and CPT2, even though non-significant, were unequal in the two groups: in the ADHD group reaction times became faster on average whereas in the control group they became slower (see Table 2 and Fig 1). There was also greater variation among the participants in CPT2 (see Table 2) than in the other conditions.

We also compared the two non-hypnotic conditions, that is, before induction and after termination of hypnosis. The difference between CPT1 and CPT4 was not significant in the ADHD group [*t*(26) = 1.15, *p* = 1.00, *d* = 0.22] but was significant in the control group [*t*(30) = 3.30, *p* = 0.008, *d* = 0.59]. The gain score analysis showed that the difference of CPT1-CPT4 was not statistically significantly different across groups [independent samples t-test, *t*(56) = -1.41, *p* = 0.163].

### Errors

A Poisson regression analysis was performed to investigate whether the ADHD and control groups differed in individual experimental conditions. The results showed that in CPT1 (likelihood ratio chi-square(1) = 2.55, *p* = 0.11) and CPT4 (likelihood ratio chi-square(1) = 0.996, *p* = 0.32) group membership clearly did not predict the number of errors committed. The respective values of the regression coefficients were naturally not statistically significant (*p*s>0.1).

Looking at CPT2 (hypnotic induction), the likelihood ratio test approached statistical significance [chi-square(1) = 3.63, *p* = 0.057], with a regression coefficient of b = -1.39 [likelihood ratio chi-square(1) = 2.97, *p* = 0.085]. The result reflects the fact that, as a group, the ADHD subjects committed 14 errors and the control subjects 4 errors in the CPT. As can be seen from Table 2, there was especially an increase in omission errors rather than commission errors in the ADHD group.

In CPT3 (hypnotic suggestion), the likelihood ratio test produced a significant result [chi-square(1) = 9.12, *p* = 0.003], with a regression coefficient of b = -2.15 [likelihood ratio chi-square(1) = 5.68, *p* = 0.017]. A subject belonging to the control group was thus expected to commit, on average, roughly only one ninth (e^-2.15^ = 0.12) of the errors of a subject belonging to the ADHD group.

To investigate whether hypnosis and hypnotic suggestions influence the number of errors committed during the individual experimental conditions, GEE analyses were performed as described in the Methods section. The analyses were performed separately in both experimental groups, and additionally in the whole group. No statistically significant effects were found (all three Wald chi-square tests for the significance of the overall effect of the predictor had *p*s>0.05).

### Hypnotizability

The mean hypnotizability measured with HGSHS:A (Table 1) was in the ADHD group 7.15 (SD = 2.52; range from 1 to 11) and in the control group 6.42 (SD = 2.36; range from 0 to 10). The difference between groups was not statistically significant (*t*(56) = 1.14, *p* = 0.26).

To investigate the influence of hypnotizability on the reaction time decrease due to hypnotic suggestions, all participants were first divided into low, LSI, (HGSHS:A 0–3, n = 6) and high, HSI, (HGSHS:A 9–12, n = 16) suggestibility groups. The difference of the mean reaction times between CPT2 and CPT3 conditions by LSIs and HSIs did not differ statistically [*t*(20) = -1.58, *p* = 0.13]. However, there was a trend towards that HSIs improved their mean reaction times more (improvement: 88.9 ms (12.9%), sd: 96.6 ms) than LSIs (improvement: 19.3 ms (2.9%), sd: 76.7 ms). Note here, that the sample size of LSI group was quite small. Second, the suggestibility was treated as a continuous variable (HGSHS:A 0–12) and its correlation with reaction time improvement with the suggestions was calculated. Pearson’s correlation r was 0.23 between hypnotic suggestibility and mean reaction time improvement between conditions CPT2 and CPT3. The correlation coefficient was not statistically significant (*p* = 0.088). In the ADHD group, Pearson’s correlation r was 0.16 (*p* = 0.43) and in the control group Pearson’s correlation r was 0.39 (*p* = 0.03). Because of the small number of the errors, we did not analyze the effect of hypnotizability on the number of errors.

## Discussion

The main purpose of this study was to investigate whether hypnosis and hypnotic suggestions influence the performance of ADHD and control participants in the continuous performance task and whether the effect is different in the two groups. There was a decrease in reaction times in both groups between neutral hypnosis and hypnosis with suggestion conditions. The performance profiles of the two groups, however, differed.

### Influence of hypnotic suggestions on reaction times

There was a statistically significant decrease in reaction times in both the ADHD and control groups from CPT2 (after induction, neutral hypnosis) to CPT3 (after suggestions) indicating an influence of hypnotic suggestions on reaction times in the attention task. The difference between CPT1 (before hypnosis) and CPT3 was statistically significant in the control group and almost significant with moderate effect size in ADHD group. There are no previous studies about the influence of hypnotic suggestion on CPT.

The results are, however, in line with previous studies with other kinds of attentional tasks. The reduction or elimination of Stroop effect after posthypnotic suggestions has been shown in several studies [21–24]. Also, the Flanker compatibility effect [44] and the Simon effect [45] were eliminated or reduced with posthypnotic suggestions in HSIs. In a preliminary study suggestions reduced McGurk effect in HSIs [28].

In these tasks, there are different kinds of conflict conditions, and suggestions shift the automatic processes to being under control. There is also preliminary data that more controlled processes, like motion perception and visual search, can be shifted to be more automatic with posthypnotic suggestions [28]. All of these tasks are, however, quite different to CPT. Our results indicate that hypnotic suggestions have an effect on reaction times in the sustained attention task CPT.

### Difference between the groups in the reaction times

In the baseline measurement, the reaction times of the control group and ADHD group did not differ. This is in line with most of the previous studies [29,35,36], although in some studies the ADHD patients have been slower [32,34] or slightly faster [33] than controls. The actual reaction times of the groups cannot be compared to other studies due to different procedures and stimuli being used in different studies

There was a statistically significant group × test condition interaction indicating different performance patterns across the groups. Thus, the reaction time profiles across CPT measurements were different in the two groups. In the ADHD group, mean reaction times were the longest in CPT1, followed by decrease in reaction times in both CPT2 and CPT3. In the control group, mean reaction times were the longest in CPT2, followed by a substantial reduction in reaction times in condition CPT3. Although the changes in reaction times from CPT1 to CPT2 were not statistically significant separately in either of the groups, there was a tendency for the reaction times in the control group to increase and for those in the ADHD group to decrease slightly. Thus, we found only suggestive, not statistical, support for our hypothesis about slowing of control participants which has been found in a previous study [37]. The tendency in different patterns is interesting. We can speculate that because the induction includes focusing of attention, the impact is different on ADHD participants because they have deficits in attention. Alternatively, the effects of relaxation on attention/reaction times might, in general, be different in ADHD patients and control subjects. There are no previous studies on the impact of hypnosis on attention in ADHD adults, thus more research is needed.

The influence of suggestions on reaction times was slightly weaker in ADHD group (reduction from CPT2 to CPT3 7.5%) than in controls (16.2%). When compared to the baseline, the difference between the groups was smaller (reduction from CPT1 to CPT3 was 9.1% and 14.0%, respectively). Thus it seems that there is no great difference between the groups but it is possible that the influence of suggestions on reaction times is slightly greater in controls. However, marked individual differences exist, especially in the ADHD group.

The comparison of conditions CPT1 and CPT3 revealed that after applying the conservative Bonferroni correction their difference was statistically significant in the control group but not in the ADHD group. However, the difference bordered on statistical significance also in the ADHD group and the effect size of the comparison was moderate. This finding can be interpreted as showing that, perhaps partly due to the smallish sample sizes, it remains a possibility that the difference was due to random variation in the ADHD group. Still, taking into account the moderate effect size, it seems at least equally plausible to consider the effect as genuine also in the ADHD group.

When examining the profiles in the Fig 1, some of the results seem a bit counterintuitive. For example in the ADHD group, the CPT2-CPT3 difference was clearly statistically significant, with the CPT1-CPT3 difference only bordering on statistical significance. The reason for this is the fact that the patterns of change differed across participants in the ADHD group. 19 (70.4%) of the ADHD participants performed faster in CPT3 than in CPT1 but eight (29.6%) performed slower. We did not find any reasons for this, e.g. the severity of the symptoms did not explain this. Further studies are warranted.

There was a difference between the non-hypnotic conditions CPT1 and CPT4 in the control group but no difference in the ADHD group. However the difference was not statistically significantly different across groups. It is possible that some posthypnotic inertia may occur [46–48] and affect the performance.

### Errors

There was no statistical difference in the number of errors in the baseline CPT task (CPT1) between the ADHD and control groups. In previous studies adults with ADHD have made more errors than controls [29,32–36]. The absence of difference in error rate between the groups in this study might be due to the low number of overall errors (0.22% and 0.06% in ADHD and control groups respectively), which in turn is probably due to the short version of the CPT task used.

In the ADHD group there was an increase in error rate after induction, but in other conditions the number of errors remained approximately the same (0.22%, 0.52%, 0.56% and 0.52% in CPT1, 2, 3 and 4). In the control group the error rate was very low in all conditions (0.06%, 0.13%, 0.06% and 0.29% in CPT1, 2, 3 and 4, respectively). According to the statistical analysis, the ADHD participants made more errors than the controls in the CPT3 condition, but the differences in the number of errors were very small, making it difficult to draw definite conclusions. There are no previous studies about the effects of suggestions with similar tasks but the results obtained here are in line with studies with other kind of attentional tasks, e.g. Flanker task [44] or Simon effect [45]. The increase in the number of errors in the CPT2 condition in the ADHD group was mainly due to the increase in omission errors. The increase of omission errors after induction has previously been found with quite a similar task in the controls [37]. However, this finding could not be supported by our data. It is possible that the attentional task has to last longer in order to reveal this possible effect of hypnosis in normal individuals.

### The effect of hypnotizability

The mean susceptibility measured with HGSHS:A was in both groups (ADHD: 7.15, controls: 6.42) in the same range as in Finnish norms (7.26; [41]), even though the controls’ score was slightly lower. The ADHD participants’ score was slightly higher than in the only existing study of ADHD patients (5.9; [49]). Taken together, the present result and the previous results suggest that the hypnotizability of the ADHD patients is in the same range as that of healthy control subjects. This result further supports the idea that it is possible to use hypnosis with ADHD patients (see e.g. [50]).

In the current study, the effect of hypnotic suggestions on reaction times compared to neutral hypnosis did not statistically differ between LSIs and HSIs. Further, there were no significant correlations between reaction time improvements in all participants. However, there was a trend for the HSIs to improve more than the LSIs, and in the control group there was also a moderate correlation between hypnotizability and improvement of reaction times. In the studies with other kind of attentional tasks, the reduction of Stroop effect [21–24], Flanker compatibility effect [44] or Simon effect [45] by hypnotic suggestions have been shown in HSIs but not in LSIs. However, in study by Raz and Campbell [25], the reduction of the Stroop effect, although in lesser extent, was also found with LSIs.

### Limitations

Our study has some limitations that should be considered when interpreting the results. Our version of the CPT took only three minutes, compared with a more usual situation when it might last for 10–30 minutes. We chose a short version of the task to avoid prolonging the hypnosis sessions unnecessarily. However, this may have resulted in fewer errors in the groups as mentioned earlier. Also, the other properties of the CPT task (e.g., visual/auditory modality of presentation, number of targets, etc) might have an influence on the subjects’ performance. It is also possible for some kind of a learning effect to occur in this kind of task. However, the profiles (see Fig 1) do not support this.

Another methodological compromise was the lack of counterbalancing between different conditions as well as not including a non-hypnotic condition in our study. This decision was made due to several reasons. There is evidence that in addition to hypnotic suggestions, also non-hypnotic suggestions reduce the Stroop effect [22]. The reverse was, however, found in the Simon [45] task, i.e. the effect was found only with hypnotic suggestions. However, there is a clear risk for confounding when the same suggestions are given in both non-hypnotic and hypnotic conditions. For example Zamansky et al. [51] noticed that subjects who had already been assessed in the hypnosis condition were less responsive to non-hypnotic suggestions (hold-back effect). Regarding the counterbalancing between conditions we know that a person may not be in a same alert state of consciousness after hypnosis has been used [46–48], i.e. a carry-over effect may occur. Also the practice effect may be different depending on which condition precedes which. We concluded that we could not balance these different effects in a within-subject design with a restricted number of clinical participants in a satisfactory way. An interesting follow-up study would be to replicate our experiment by using e.g. relaxation instead of hypnosis but still keeping all the suggestions exactly the same.

In this study, hypnotizability was measured once by using HGSHS:A. An individual assessment in addition to a group variant would have clearly given a better estimate of the participants’ hypnotizability. However, the use of the previously popular Stanford Hypnotic Susceptibility Scale, Form C (SHSS:C) [52] includes items (e.g. dream, age regression and ammonia), which have lately been heavily criticized [53,54]. In recent studies only modulated versions of SHSS:C have been used which clearly compromises the value of the data they provide. Due to these reasons we did not choose to use SHSS:C especially keeping in mind that almost half of our participants were clinical patients.

It is also possible to explain the result by referring to demand characteristics produced by the hypnotic induction and suggestions. We tried to reduce this risk by measuring reaction times, since it is difficult to modulate them, at least intentionally, in tasks that require fast responding to a challenging attentional task.

### Clinical Implications

Oakley and Halligan [11] have suggested that it is worth to study hypnosis also as a neurocognitive rehabilitation tool. Psychosocial treatments have proven to be beneficial in treating adult ADHD. The most studied treatment is CBT [55–59]. The focus on CBT treatments is on compensatory strategies, altering dysfunctional thoughts and attitudes, and improving metacognition. In one existing study in adults with ADHD, hypnotherapy was as effective as CBT [49] but there was better long-term outcome for hypnotherapy than for CBT [60].

These previous studies were not directly targeted to the core cognitive symptoms of ADHD, such as problems of attention or working memory. In this study, we did not find any effects of hypnotic suggestions on the attention errors, probably because of the short attentional task. We, however, did find that hypnotic suggestions influenced the reaction times in a sustained attention task, so it seems that it is possible to influence the cognitive performance of ADHD patients by hypnotic suggestions. Despite the limitations of this study and the caution that must be exercised when interpreting the results, it is worthwhile in further research to study more the possibilities of hypnosis and hypnotherapy on the attention deficit and other core deficits of ADHD patients.

## Conclusions

This study indicates that hypnotic suggestions have an influence on the reaction times in a sustained attention task. This effect was found in both adults with ADHD and normal control participants. However, this result has to be verified in further studies also with non-hypnotic suggestions. This study, together with the pilot study of hypnotherapy in adults with ADHD [49,60], suggests that with hypnosis and hypnotherapy it is possible to influence the problems on ADHD adults.
